# A “plan bee” for cities: Pollinator diversity and plant-pollinator interactions in urban green spaces

**DOI:** 10.1371/journal.pone.0235492

**Published:** 2020-07-15

**Authors:** Benjamin Daniels, Jana Jedamski, Richard Ottermanns, Martina Ross-Nickoll

**Affiliations:** Institute for Environmental Research, RWTH Aachen University, Aachen, Germany; RMIT University, AUSTRALIA

## Abstract

Green infrastructure in cities is considered to serve as a refuge for insect pollinators, especially in the light of an ongoing global decline of insects in agricultural landscapes. The design and maintenance of urban green spaces as key components of green infrastructure play a crucial role in case of nesting opportunities and for foraging insects. However, only few research has explored the impact of urban green space design on flower visitor communities, plant-pollinator interaction and the provision of the ecosystem service of pollination in cities. We investigated the abundance and diversity of pollinator communities in different urban park types in designed, standardized vegetation units, linked the visitation rates to the structural composition of the park types and derived indices for implemented pollination performances. The study was performed in two different structural park elements, flower beds and insect-pollinating trees. To gain a comprehensive understanding of the interaction between plants and pollinators, we calculated a plant-pollinator network of the recorded community in the investigation area. Visitation rates at different park types clearly showed, that the urban community gardens in comparison to other urban park types had a significantly higher abundance of pollinator groups, comparable to results found on a rural reference site. Tilia trees contributed significantly to the ecosystem service of pollination in investigated green spaces with a high supply of nectar and pollen during their flowering period. Calculations of pollination performances showed that recreational parks had comparably low visitation rates of pollinators and a high potential to improve conditions for the ecosystem service of pollination. The results indicated the strong potential of cities to provide a habitat for different groups of pollinators. In order to access this refuge, it is necessary to rely on near-natural concepts in design and maintenance, to create a wide range of flower diversity and to use even small green patches. Based on the findings, we encourage an integrated management of urban free spaces to consider parks as key habitats for pollinators in anthropogenic dominated, urban environments.

## Introduction

Flower visitors are ecological keystones as they pollinate 78–94% of all wild plants [1]. Their diversity builds the foundation for the maintenance and support of services and functions in ecosystems [2, 3] and improves resilience in socio-ecological systems [4]. Insect pollination is not only a crucial ecosystem service, but a key process for numerous ecosystem services. It supports the preservation of plant and animal populations as well as crop yields [5]. Many plant communities depend on pollination by insects, agricultural economy can only receive a sufficient crop yield by insect pollination [6]. The honeybees (*Apis mellifera*) account for the largest proportion of pollinators worldwide in the agricultural landscape [7], they help to pollinate a third of our food [8] and assure food industry benefits by honey production. Wild insects perform, due to their pollination preferences, in supplement to honey bees and not as a substitute [9]. Klein, Vaissiere [10] found that about 75% of crop species need insect pollination. The global economic value of animal pollination was estimated to be 153 billion Euros [11]. Moreover, the protection of species richness of flora and fauna has an intrinsic value, derived from a pathocentric perspective of animal ethics, as well as from a natural-ethical motivation [12]. Therefore, a comprehensive understanding about the relationship between species abundances, diversity of pollinators and the ecosystem function of pollination is needed. This will enable to link species richness with a respective function and service, which is often lacking in biodiversity and ecosystem service research in general [13, 14] due to limited knowledge [3].

A multitude of anthropogenic stressors lead to a dramatic global decline of pollinating flower visitors, in terms of insect biomass, species richness and number of individuals [7, 15, 16]. Reasons are diverse and frequently discussed throughout the last years [17]: In agricultural landscapes, pollinators have to cope with a multitude of pesticides, which may occur as combined exposure events over time and space [18, 19]. Beside this, the loss of structural diversity and habitats as well as the degradation and isolation of habitats [20] as a direct effect of monoculture cultivation in agricultural landscapes affect the abundance and biodiversity of pollinators [21, 22]. Additionally, soil sealing due to increasing urbanization [23–25], climate change [16] and the spread of non-native species affect pollinator communities [26]. For cultivated honeybees, an increase in parasite infections, especially by *Varroa destructor* [8] or American foulbrood, and improper apicultural practices lead to an increase of bee colony mortality [27]. The interaction of these drivers leads to a shift and an endangerment of pollinator communities [28], also having far-reaching and adverse consequences on economic, climatological and cultural perspectives. On an ecological level, the loss of insects will have serious consequences for other animal groups. If insect pests occur on a massive scale, there is a risk that parasitic insects will no longer be present as opponents. Moreover, studies already showed clear population decreases in insectivorous bird species, which are lacking food resources, correlated with the presence of pesticides in surface water [29].

Urban environments contain a variety of these threats and stressors to pollinators. Nonetheless, studies have shown that urbanization can also raise positive effects and opportunities for pollinator communities [30, 31] by supporting and supplying resources for food, nests and hibernation. Environmental conditions like the absence of pesticides in cities leads to significantly greater harvests of honey per year in urban bee hives in contrast to land hives in agricultural landscapes [32, 33]. Furthermore, cities generally count on a higher flower diversity and density on urban green spaces than in rural areas [34, 35], holding new habitat opportunities and food resources [36]. Composition of flowering plants as food sources affects the richness and diversity of pollinators [37]. Especially the richness and long vegetation periods of flowering blossoms in flower beds of cities attract a large number of pollinators [38]. Because of co-evolutionary developments of flower blossom and pollinator morphology, the interaction has developed into a finely adjusted system. In many cases only distinct groups can form a successful symbiosis. This functional traits of plants are defined as *“pollination syndrome”*, which integrates morphological properties of blossoms and pollen vectors. The classification is based on the *flower class* according to Müller [39] and the *flower type* of Kugler [40]. It contains information about the pollination process (e.g. by water, wind or animals) and the preferred animal pollinator (biotic pollination) of a respective plant due to the flower structure. Interaction networks were established as a valuable instrument to investigate these relationships [41, 42]. Two different perspectives can be adopted when observing plant-pollinator interactions. On the one hand as a food source and livelihood for the endangered flower visitor group, on the other hand as a key ecological property for plant distribution and conservation. The ecosystem service of pollination can thus be seen from two sides of the coin. This duality of observation based on the dynamic interaction between plant and pollinator is also reflected in plant-pollinator-interaction networks [41]. Due to these different preferences of flower visitors and plants it is crucial to design green spaces with diverse pollination syndromes in order to provide food sources for miscellaneous pollinator groups [37].

Therefore, a shift in pollination research for urban areas can be noticed. The city is not able to replace a pristine habitat of each pollinating insect, but cities can contain alternative refuges [31, 36]. This, however, requires a suitable structural design and maintenance of urban green infrastructure, and in particular of urban green spaces as core areas of green infrastructure in cities [43, 44]. Urban green spaces are a multitude of different open spaces in cities, such as parks, cemeteries, community gardens etc., which have a predominant proportion of vegetation structures (also called *park types* in this study). Knowledge about the relationship between green space structure, plant and pollinator richness and diversity, and the link to an adequate provision of the ecosystem service of pollination in cities is urgently needed to improve future management strategies [14]. In this research study we aimed to assess different park types, their management concepts and the resulting structural properties with regard to the abundance and diversity of plant pollinator groups as well as the implementation of the ecosystem service of pollination. To do this, we followed a multistep procedure to answer the following research questions:

1. Comparison of different park types: In a first step, an investigation was carried out in order to quantify to what extent park structure affect the recorded pollinator community. Therefore, a comparison of pollinator communities between different parks and park types (depending on their underlying management concepts) was conducted. We installed structurally identical flower beds (“designed beds”) in eight different parks and a rural reference site. Flower visitors were recorded according to a standardized methodology.
2. Comparison of the composition and abundance of flower visitor groups between designed beds and typical public flower beds within the same green spaces: We aimed to detect differences in the pollinator communities in order to obtain a comprehensive pattern of the pollinator community at a site and for the entire green spaces. To get insights into the plant-pollinator interactions and dynamics, we used the observational data from public beds to calculate plant-pollinator networks to draw conclusions about a pollinator-friendly design of urban beds.
3. Relevance of insect-pollinated Tilia trees (*Tilia* spp., linden trees) for the ecosystem service of pollination: Results of the pollinator observations in flower beds were compared to investigations on insect-pollinated city trees. This provides information on the extent of the influence for different park structures regarding the provision of pollination in urban green spaces.
4. Estimation of overall pollination performances in park types: In order to assess the total provided ecosystem service of pollination in different park types, we developed and calculated a *pollination estimator* index for flower beds and trees based on our recorded data. The *pollination estimator* provides information about the actually realized performance of the park types and identifies potential for improvement with regard to pollination.
5. Recommendations for planning and designing procedures of urban green infrastructure: Based on the results we derived recommendations for action regarding the structural design and maintenance of urban green spaces. This leads to a discussion about the integration of pollination services into an integrative management concept for green spaces in cities.

## Materials and method

### Investigation sites and park type categorization

**Fig 1:**
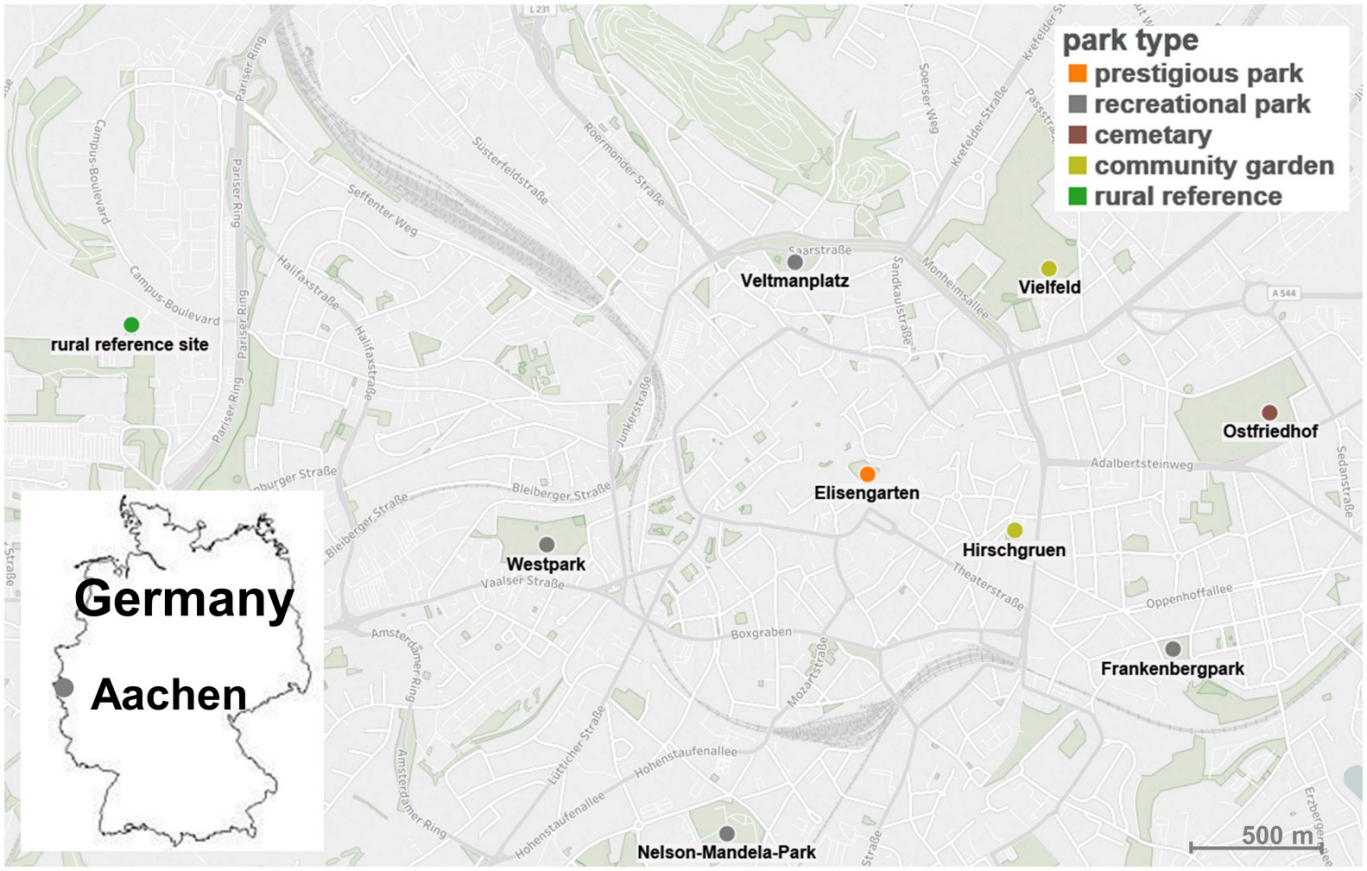
Investigation sites and park types located in the city of Aachen, Germany.

The study took place in the city of Aachen, Germany, a medium-sized town (250.000 citizens) in Western Germany close to the borders of the Netherlands and Belgium (Fig 1). We chose eight green spaces situated in an urban surrounding and one rural reference as investigation sites (Fig 1). All urban green spaces are parks and park-like designed and maintained free spaces. The green spaces are free to use and open to citizens and visitors of the city. In addition to differences in size and location of the green spaces, the parks also differ in the various guiding and management concepts developed by the municipal authorities. Therefore, we categorized the green spaces into four different park types with regard to their underlying concept of maintenance which directly affects the vegetation structure of the green space. The park type categorization system is given in Table 1.

**Table 1:** Green spaces and associated park types of the study. Visual illustrations of the park types are shown in the Supporting information, S1 Table.

Park type	Park name	location decimal degree (latitude, longitude)	Description
**prestigious park**	Elisengarten	50.774216, 6.086301	representative green space in the very center of the city
**recreational park**	Westpark	50.771754, 6.0686	central green spaces within city quarter/district recreational function for citizens
	Nelson-Mandela Park	50.761661, 6.078525	
	Frankenbergpark	50.768104, 6.103168	
	Veltmanplatz	50.78163, 6.082276	
**community garden**	Stadtgarten / Vielfeld	50.781392, 6.096308	recreation, gardening and social exchange extensive management
	Hirschgruen	50.772243, 6.094444	
**cemetery**	Ostfriedhof	50.77636, 6.108554	oldest cemetery in the city, old and lush vegetation structures
**rural reference**	-	50.779433, 6.0456099	located in a rural-urban, agricultural landscape, 2.7 km distance from the city center

The pre-categorization of the parks into finite park types was conducted in order to identify differences in the pollinator community among differences in park use, design and structure. The identified park types differ in their maintenance and utilization concepts for citizens and, consequently, in their structure regarding the composition of the single structural park elements [45].

The *Elisengarten* (representative park type) is a small park (about 8000 m^2^) in the very center of the city, close to major historic attractions such as the market square, the cathedral and the Elisenbrunnen, a large building with a thermal fountain. Typically for centrally located parks in cities, the Elisengarten was designed primarily as a representational free space by the planning authorities. It should support a positive external appearance of the city. The main focus of the local authority is put on the quality of stay for citizens and tourists. This is supported by decorative plants, and a maintenance concept which includes a high frequency of maintenance works and horticultural activities. The high level of gardening maintenance and the very strict trimming of plants leads to a highly structured appearance of this central park.

The recreational parks *Westpark*, *Nelson-Mandela Park*, *Frankenbergpark* and *Veltmanplatz* are all characterized by the fact that they serve as popular, central meeting places of the respective districts in the city. They all are equipped with various and frequently used leisure facilities (barbecue area, playground, basketball court etc.).

Community gardens *Vielfeld*, located in the Stadtgarten, and *Hirschgruen* are collectively managed green spaces with a relatively large percentage of vegetable and flower beds maintained by volunteers. They are open to the public, anyone can use the gardens and participate in its maintenance. In terms of surface area, however, the community gardens are the smallest of the considered green areas (*Hirschgruen* with 1,810 m^2^ and *Vielfeld* with 2,360 m^2^). The free design opportunities for citizens lead to a large number and diversity of different structural park elements. In addition to many ornamental flower and vegetable beds, intensive care of the shrubs and meadows is deliberately avoided (e.g. mowing of lawns generally only twice a year). Maintenance of the green space is based on ecological principles. Plant protection products such as herbicides or insecticides are not applied and a seed mixture of plants from typical regional meadows was used for the planting of the large central lawn.

The *Ostfriedhof* is the oldest still used cemetery area in the city center. Due to its age of about 220 years, this green area is lush with mature trees and shrubs. A relatively large number of flower beds are maintained and regularly renewed.

As a *rural reference*, an agricultural landscape site was chosen, situated in the rural-urban fringe of the city of Aachen. It is located 2.75 km in linear distance from the very city center. In the surrounding of this extensively used meadow different vegetation structures of the open country, such as meadow orchards, pastures, groves and fields are situated. In comparison to the investigation sites in the city center, the degree of sealing is significantly lower.

### Recorded data: Plant and pollinator community

A three-pronged approach was chosen for this investigation:

First, the pollinator communities of park types were compared to each other directly by designing, planting and recording small-scaled, identical flower beds (*study beds*) as food source for pollinators. This enables us to assess the abundance of flower visitors in all different park types under equal conditions and with the identical supply and attraction of plants. Flower beds in various parks usually have a different plant community and therefore—in terms of pollination syndromes—different conditions to attract present flower visitors.

Second, typical *public beds*, that contain a selection of flowering plants with a mixed setup of representative pollination syndromes for each park were observed in addition to the *study beds*. The typical pollinator community structure and pollination interactions in park types were analyzed by applying this setup. We also used the data to perform a plant-pollinator interaction analysis for the community in the investigated parks.

In a third step, we recorded data for Tilia tree pollination to estimate the total number of flower visits and pollination performance in the investigated parks.

#### Flower bed preparation and plant community of designed beds

Eight inner city green spaces and one rural reference site were equipped with *study flower beds*, supported by the municipal park authorities of the city of Aachen.

**Fig 2:**
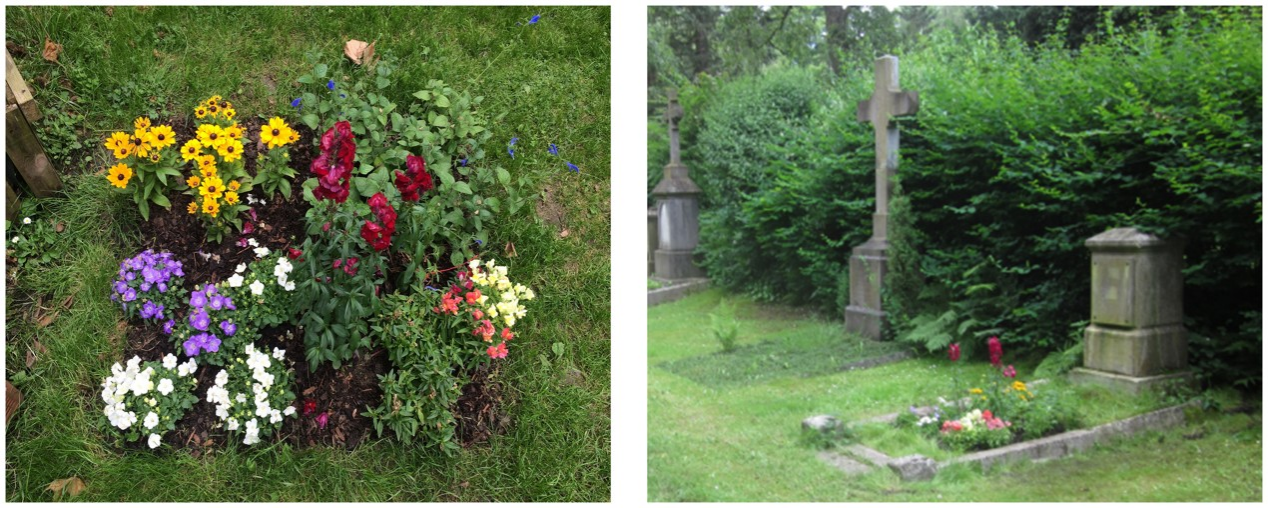
Arrangement of standardized plants in the designed study bed (left) and example of an investigated study bed (site: Cemetery *Ostfriedhof*).

We chose the investigation patches of one square meter and planted the flower beds in the same way to standardize our experimental design and identically attract flower visitors on all study sites. It was ensured that the specific site within the park was sufficiently exposed to sunlight during the day. The turf was removed, soil was dug up and the gaps for the plant root balls was filled with granulated fertilizer for a sufficient blossom formation during the summer season. By selecting four different plant species, each quarter of the square meter was filled with different plant species. Plants were not planted randomly within the surveyed square meter to ensure that pollination syndromes were correctly assigned to visiting pollinators (Fig 2).

The study beds were planted with five plants of *Campanula carpatica* (Campanulaceae) as a bee syndrome, *Antirrhinum majus* (Plantaginaceae) as a bumblebee syndrome, *Rudbeckia hirta* (Asteraceae) as a generalist syndrome and *Salvia patens* (Lamiaceae) as a merged pollination syndrome for Hymenoptera (Table 2).

**Table 2:** Plants and associated pollinating service of the designed study beds.

plant species	family	associated pollination syndrome
***Rudbeckia hirta***	Asteraceae	generalists
***Salvia patens***	Lamiaceae	Hymenoptera
***Campanula carpatica***	Campanulaceae	bees
***Antirrhinum majus***	Plantaginaceae	bumblebees

The plant species are suitable for the most common groups of pollinators. We took into account that certain groups of flower visitors are more frequent than others and that they might behave individually in terms of potential specialization (e.g. flower constancy).

The selection was especially adapted to the groups of bees (e.g. honeybees, solitary bees and bumblebees) and hoverflies (Syrphidae). We assumed bees to be the most abundant flower visitor group. Therefore, the four plant species were chosen to meet the requirements for solitary bees, honeybees and bumblebees. A syrphid-specific flower was not selected because most species of Syrphidae were assumed to be more or less generalistic regarding their flower preferences. A typical plant with syrphid-adapted pollination syndrome like umbellifer (Apiaceae) was not manageable in the context of a small decorative flower bed (due to their strong growth and their non-ornamental appearance). However, since syrphids are known to get attracted by other disk flowers, we decided to select a representative of the daisy family (Asteraceae, *Rudbeckia hirta*) as a substitute. Asteraceae are also able to attract pollinators like butterflies and beetles.

#### Plant community of public flower beds and selection of Tilia trees

**Fig 3:**
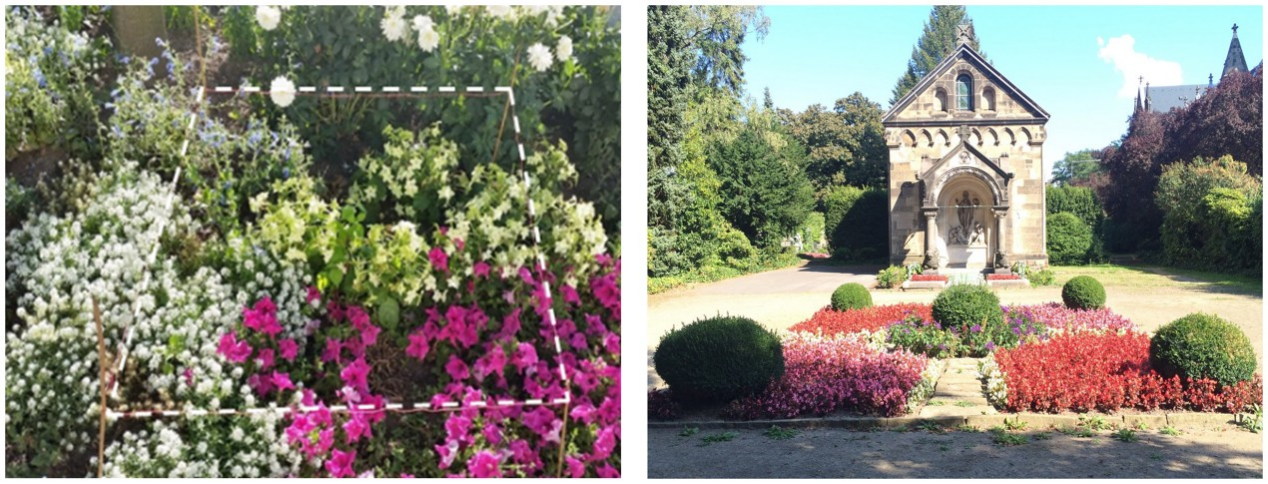
Defined investigation area within a public bed with marked surveyed area of one square meter (left, site: Representative park *Elisengarten*) and example of a surrounding area (right, site: Cemetery *Ostfriedhof*).

In addition to the designed *study beds*, a typical square meter of existing *public flower beds* of each study site was selected for observation (Fig 3). This was not the case for the *Nelson-Mandela Park*, *Veltmanplatz* and the rural reference site, because no public flower beds were situated in these sites.

In public flower beds a typical, representative part of the plant community with pollinator attracting flower compositions was chosen. The recorded investigation patch in the public bed of a green space was always the same for every repeated observation.

The public flower beds of the representative park *Elisengarten* and the recreational park *Frankenbergpark* were dominated by Hymenoptera syndrome (Lamiaceae). The flower bed of the *Westpark* had various pollination syndromes like bumblebee flowers, generalist flowers and a prevalence of the species *Cleome spinosa* which is known to produce a lot of nectar (uncategorized pollination syndrome). Public flower beds of the cemetery Ostfriedhof were dominated by *Begonia semperflorens* which is known to produce only a small amount of nectar but pollen (uncategorized pollination syndrome). The public beds of community gardens *Hirschgruen* and Stadtgarten/*Vielfeld* were mainly planted with generalist pollination syndromes (Asteraceae) by the park authorities. A detailed list of all recorded plant species on the public flower beds is given in Supporting information, S3 Table.

**Fig 4:**
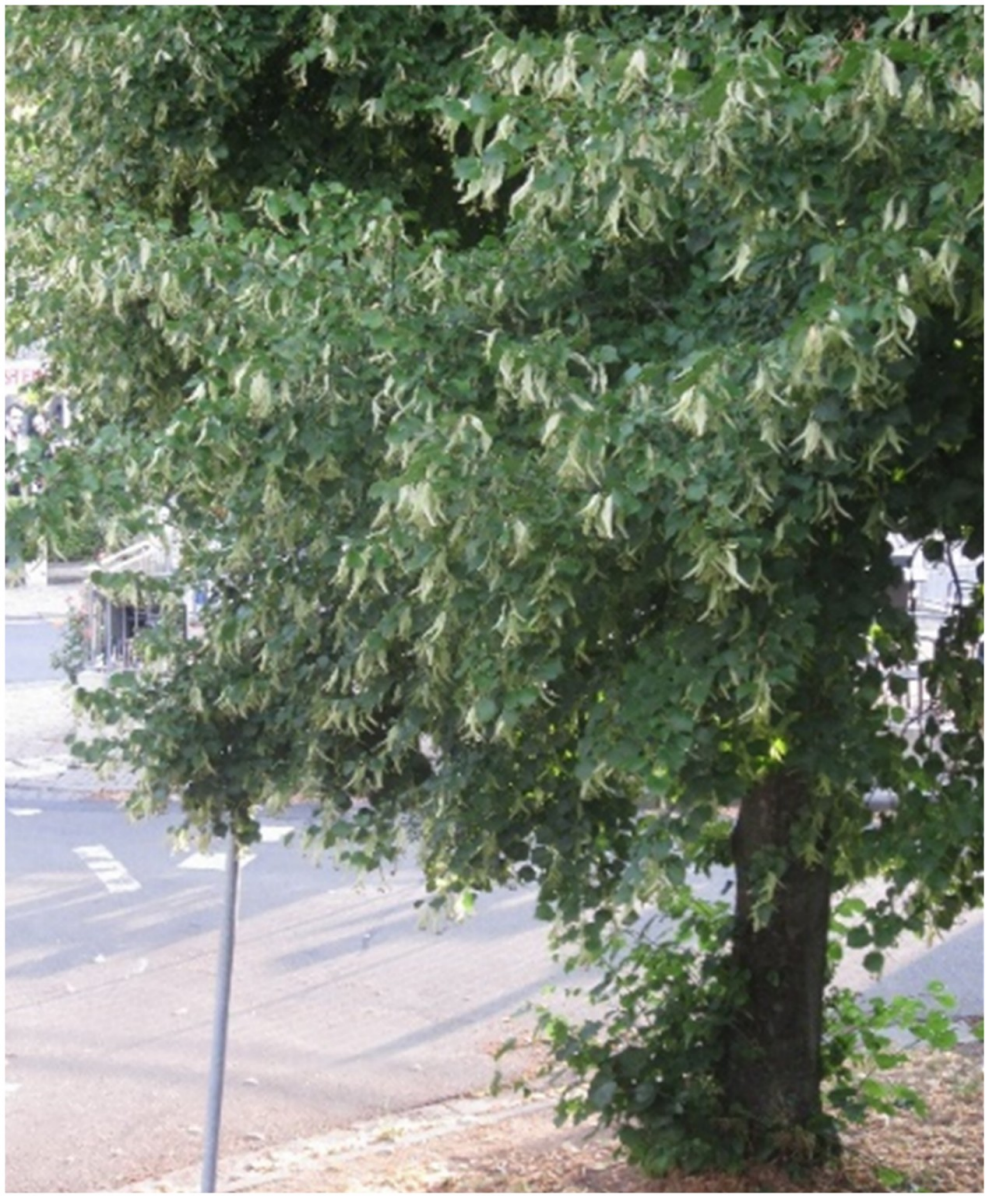
Branches of a flowering Tilia tree in the city of Aachen, July 2016.

In addition to the beds, insect-pollinated trees were selected in the investigated parks. Due to their dominant abundance in Aachen, similar to most medium-sized or large cities in Germany [46], only trees of the genus Tilia (*Tilia cordata*, *Tilia platyphyllos*, *Tilia tomentosa*, *Tilia dasystyla*; Malvaceae) were chosen in this study (Fig 4). Tilia trunks have a high regenerative capacity and a resulting high stability. They are tolerant towards hot summers and drought. Therefore, Tilia trees are suitable for the various external stressors that affect urban trees (immission load by road traffic, road salt, mechanical damage, high outside temperatures, soil compaction, etc.). Tilia trees of the investigation sites were identified and one m^3^ of the treetop-green volume surveyed. The number of blossoms within the m^3^ were counted and data included into the study (see sampling method).

#### Pollinator community: Recorded pollinators and morphogroups

The insects were monitored by observation in order to have as little invasive effects on the community as possible (see sampling method). Depending on the question under consideration, we classified the recorded individuals into different groups of insects:

Eight morphogroups of flower visitors were differentiated in public beds for plant-pollinator analyses; small wild bees (mainly *Lasioglossum* and *Halictus* species), mason bees, leafcutter bees, carder and resin bees (all members of the family Megachilidae), large solitary wild bees, honey bees (*Apis mellifera*), *Bombus terrestris*, bumblebee species, *Episyrphus balteatus* and hoverfly species. Less frequently recorded pollinators such as butterflies and beetles were counted and analyzed as single findings (S2 Table).

The three species *Apis mellifera* (bee), *Bombus terrestris* (bumblebee) and *Episyrphus balteatus* (hoverfly) are the most frequent representatives with an outstanding position in their respective groups. *Apis mellifera* and *Bombus terrestris* provide the largest share of pollination service, *Episyrphus balteatus* as a frequent representative of the group of hoverflies does not only support pollination, but also provides a nature-based pest control service. Their larvae feed on aphids. The three species are comparably easy to identify and were therefore recorded separately. Recording the individuals of *B*. *terrestris* is a challenging task though, especially in cases when yellow stripes were as bright as known from *Bombus lucorum*. However, most individuals can be correctly assigned to this species by some practice, due to the darker coloring of the yellow stripes of bumblebees. Furthermore, the large individuals of Megachilidae were easy to identify by their ventral pollen-carrying structure (scopa) when it was filled with pollen grains. We used this more detailed categorization system to conduct the plant-pollinator-interaction analyses.

**Fig 5:**
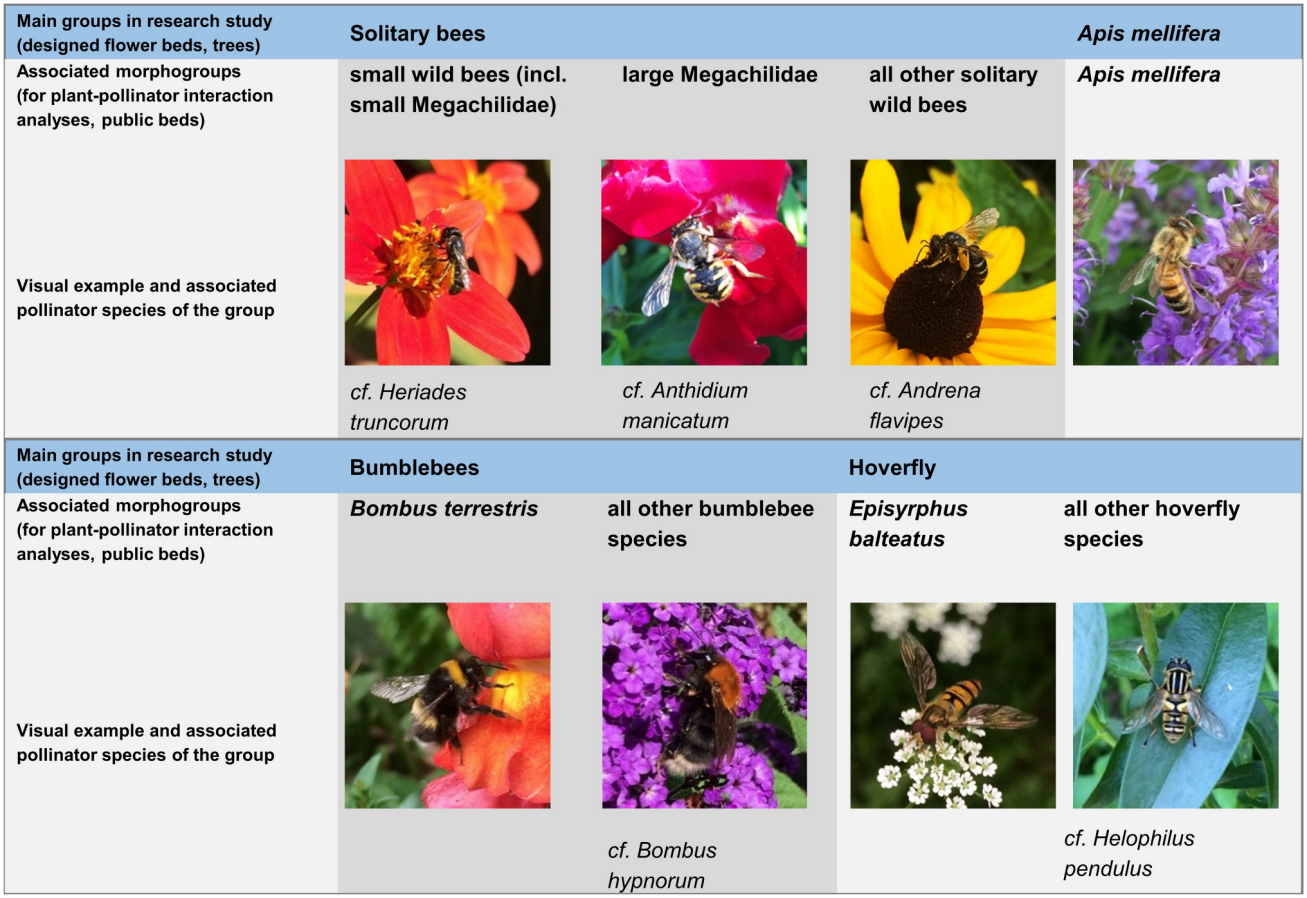
Representatives of differentiated pollinator groups.

The abundance analyses of the general survey (designed flower beds and Tilia trees) were carried out with five main groups in which the morphogroups were merged (Fig 5). The evaluation and comparison of the overall abundance appeared to be most meaningful at this level of differentiation. For the detailed classification of pollinator species into morphogroups and classes within the investigation, please find the respective table in the Supporting information (S2 Table).

### Sampling method

The performed study design and sampling method was tested and adjusted in preliminary studies in 2014 and 2015 (see Supporting information, S4 Table). From May to August 2016, in the seasonal peak period of flowering and the highest activity of flower visitors (according to the preliminary investigations), the observations were carried out on each site and patch. The observations on Tilia trees were performed within this time during the flowering period of Tilia between June 21th and July 15th.

All measurements were carried out between 10:00 a.m. and 5:00 p.m. in randomized repetition. Care was taken to ensure that the environmental conditions were as constant as possible. The tests were only carried out in sunny and not windy conditions. All environmental variables (temperature, wind, cloudiness) were recorded during the measurement.

A pollinating insect entering the study plot to visit a blossom during record time of 30 minutes (= plant pollinator interaction) was counted and classified into morphological groups. No insect was removed from investigation sites, morphogroups were counted by observation. In general, determination at species level would be more meaningful from an ecological point of view. Without catching individuals, however, reliable species identification cannot be assured. Quantitative studies on pollinator activity in the field are often and successfully carried out on morphogroup-level to maintain the balance between intrusiveness and accuracy [25, 47]. For each visit the identity of the visited plant species was recorded.

In a time period of 30 minutes all pollinators visiting the respective square meter were classified by morphogroups and counted following the Eq (1)

$$V_{group}=\frac{no.\;of\;recorded\;individuals}{dest.\;*\;30\;min}$$ (1)

with

*V*_*group*_: visitations of a specific pollinator species or morphogroup per record and park type

*dest*.: “destination”, visitation of plant species, flower bed [m^2^] or treetop (tree crown) [m^3^].

The parameter *dest*. was adjusted according to the respective analysis. The comparison of pollinator groups in flower beds was conducted for visitation rates per selected bed (1 m^2^) and in Tilia trees per one m^3^ treetop; for plant-pollinator interaction analysis, visitations were recorded on plant species or functional trait group (*pollination syndrome*).

It was differentiated if flower visitors only made a reconnaissance flight (used for overall community evaluation) or if they visited the blossoms as a precondition for pollination (used for plant pollinator interaction network analysis). In both cases it was noticed which plant species was approached.

### Statistical data analysis

All statistical analyses were performed using the software R (3.1.1) with R Studio (1.0.136). Data were pre-tested on normal distribution and homoscedasticity of variances with Shapiro-Wilks and Levene’s tests.

The comparison of differences in pollinator group visitation rates between different types of parks was conducted by performing a Kruskal Wallis test for non-normally distributed data followed by a Dunn post-hoc test for multiple comparisons. Multiple testing was performed with a sequential Bonferroni-Holm correction of the familywise error-rate alpha. A comparison of the flower visitor composition was conducted by a multivariate hierarchical cluster analysis (R package “vegan” version 2.4–3 [48]; Bray-Curtis dissimilarity index, algorithm “complete”).

To compare the two different types of investigated beds (designed study beds vs. public flower beds) in each green space pairwise Dunn tests were performed.

In addition, we carried out an analysis of the bipartite plant-pollinator visitation network for public bed data. We used the R package “igraphs”, version 1.1.2 [49] for network visualization and applied the R package “bipartite” version 2.08 [50] for plotting matrices, bipartite graphs and calculating network metrics.

Due to the diverse range of flowering plants, a more meaningful network could be expected for public beds compared to the designed study beds. The network complexity may not have been adequately represented with this data. Therefore, only the recorded data set of all public beds was used for network analyses. Moreover, a classification of the pollinator groups was applied which is slightly more detailed than in the overall analysis. The class "others" was differentiated into groups of pollinators according to experts' visual observation and judgement. Due to the relatively low number of counted visits in the group “others”, this differentiation did not make sense for any other analyses. An overview of differentiated groups is shown in the Supporting information, S2 Table.

In case of plant pollinator network analyses, recorded data of blossom visitations in the survey were summarized in a cross table for all pollinator groups and plants in public beds. Data of the matrix were normalized by correction factors, in order to consider the different numbers of replicates for the tested public beds. These imbalances in the frequency of monitored public beds and their plant species was corrected by multiplying the number of visitations with a specific factor for each plant species. This correction factor is given by the quotient of the number of replicates of the most surveyed site (Stadtgarten/Vielfeld, n = 14) divided by the number of replicates of the site the respective plant species is located. The correction factor was between 0.52 and 1.75 (Supporting information, S3 Table).

From the balanced cross table an incidence matrix was converted and applied to generate bipartite graphs. A circle layout was selected for the network illustration, the size of the vertices (= plant and pollinator species) illustrates the frequency of species in observed interactions, the edge widths reflect the numbers of interactions between respective plant and pollinators.

### Pollination performance: A brief prediction

In addition to the evaluation of the abundance and diversity of pollinators, we extrapolated the park type performances regarding the supply of the ecosystem service of pollination. This leads to rough estimations of the extent to which different structural elements (beds and insect-pollinated trees) contribute to the service of pollination, extrapolated by total pollinator visitation rates of flowers in different park types.

For this purpose, the surface areas of all flower beds and green volumes of Tilia trees in the investigated green spaces were measured. These spatial data of the structural park elements were used to extrapolate the total mean visitation rates for all Tilia trees and flower beds in the respective green spaces in 30 minutes (under given, constant environmental conditions of our investigations), according to Eq (2)

$${total\;visitations}_{flower\;bed\;/\;linden\;tree}=Mean(\sum V_{group})*spatial\;reference$$ (2)

with

*spatial reference*: surface area [m^2^] for flower beds or treetop volume [m^3^] for Tilia trees.

The mean total visitations are calculated for a time period of 30 minutes on the total respective park element (flower bed or Tilia trees) of a park type. The extrapolated visitations for total beds and trees are therefore only predictions to provide a rough comparison of the magnitude of pollination. These extrapolations are labelled in the result section ("*estimated*") to avoid confusion with recorded data.

By using these data of flower visitations as indicator metrics for the provided ecosystem service of pollination, we are able to estimate the performances of the park types regarding the ecosystem service of pollination. For this, a *pollination estimator* for all investigated park types was derived by normalizing the visitation rates in the structural park elements per area of the respective park type (3).

$${pollination\;estimator}_{flower\;bed\;/\;linden\;tree}=\frac{{total\;visitations}_{flower\;bed\;/\;linden\;tree}}{park\;type\;area\;[m^{2}]}$$ (3)

The *pollination estimator* illustrates the mean visitations of all flower beds or Tilia trees per m^2^ park area in 30 minutes.

## Results

### Overall characteristics of the dataset

We conducted a total of 187 records during the course of the investigation on study beds and public beds. In addition, we carried out another 112 field records in Tilia trees. Throughout the investigation, 7723 interactions between pollinators and plants were observed. A detailed distribution of all recorded samples for park types and structural park elements is shown in Table 3.

**Table 3:** Investigated park types, structural elements of parks and number of samplings (s = number of different investigated structural units, different beds or trees).

Park type	Structural elements	Number of records*	N total
Representative park	study bed (s = 1)	13	26
	public bed (s = 1)	13	
Recreational park	study bed (s = 4)	50	170
	public bed (s = 3)	35	
	tree (s = 12)	85	
Community garden	study bed (s = 2)	27	52
	public bed (s = 1)	13	
	tree (s = 2)	12	
Cemetery	study bed (s = 1)	13	41
	public bed (s = 1)	13	
	tree (s = 2)	15	
Rural reference	study bed (s = 1)	10	10
		**N** =	**299**

*major differences in number of investigated structural units per structure type mainly due to no occurrence of these structural elements in the respective park types

### Comparison of flower beds (between parks)

**Fig 6:**
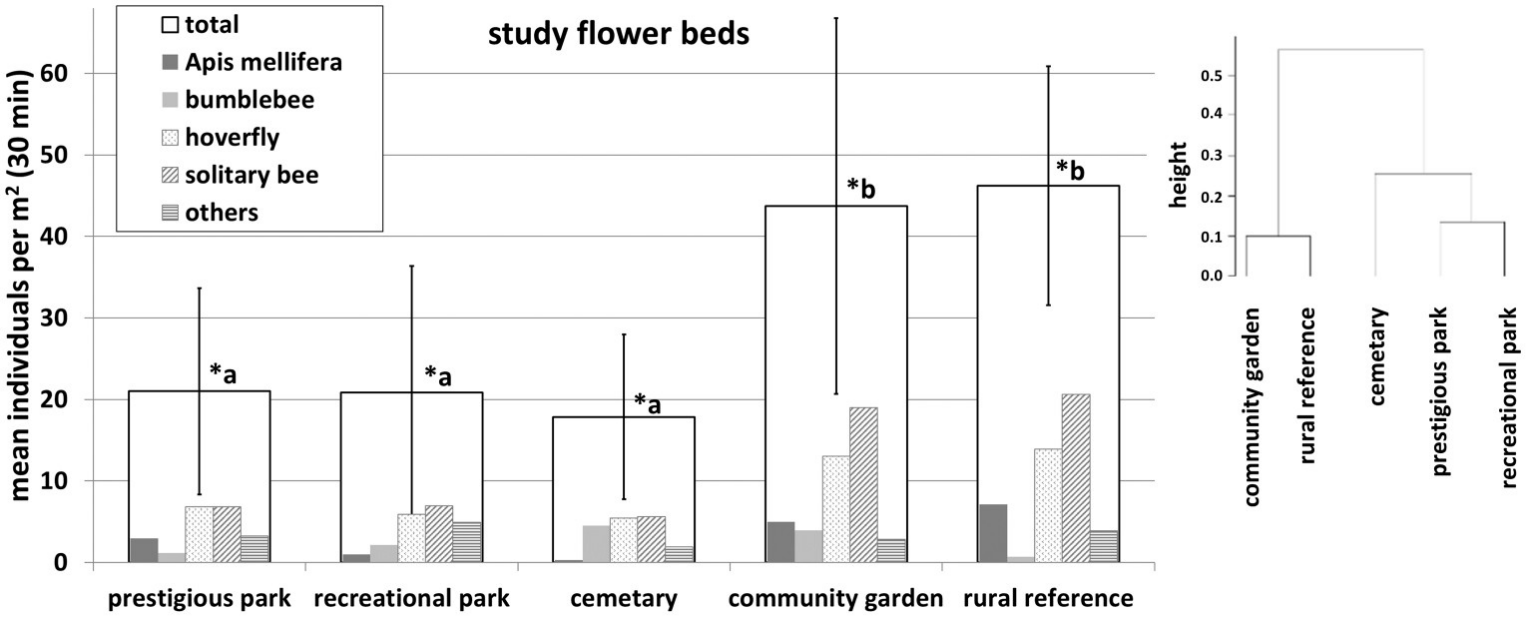
Mean number of recorded flower bed visitations for every green space type. From white to black bars: Total visitations, *Apis mellifera*, bumblebees, hoverflies, solitary bees and others. Bars with the same letter show no significant differences (p> 0.05), different letters indicate a significant difference (p< 0.05). In addition, an agglomerative cluster analysis was performed with untransformed visitation means (right figure).

The recorded numbers of visitations per m^2^ in 30 minutes for total pollinators and pollinator groups are shown in Fig 6.

The mean number of recorded insects in the rural reference site was 46.2±14.6 within 30 minutes. For urban green spaces, a comparable amount of visitations was only found in community gardens (43.7±23.0 per 30 minutes). The mean number of insect visits in the representative park Elisengarten, the recreational parks and the cemetery were significantly lower, a mean visitation rate of 21.0±12.6, 20.8±15.5 and 17.8±10.1 was recorded respectively.

It is striking that especially wild bees and syrphids show a lower amount of bed visitations in urban parks compared to the rural reference site. The mean number of solitary bees was between 5.6 (cemetery) and 6.9 (recreational park) visitations, for hoverflies between 5.5 (cemetery) and 6.8 (representative park). The rural reference showed mean visitation rates of 20.6 (solitary bees) and 13.9 (syrphids) for these insect groups. However, in the urban community garden wild bees (19.0) and syrphids (13.0) were recorded in comparable amounts as found at the fringe of the city.

The colonizing species *Apis mellifera* and *Bombus terrestris* were only recorded with small visitation rates within the urban study beds: for honey bees we observed mean rates between 0.3 (cemetery) and 5.0 (community garden) individuals, for bumblebees between 1.2 (prestigious park) and 4.5 (cemetery) individuals per plot and record.

The multivariate cluster analysis illustrated the particularly high consistency in the abundance and composition of the pollinator community in community gardens and the rural reference. A differentiation between the pollinator communities of these investigation sites and the cemetery, the prestigious park and the recreational park can be shown. Prestigious and recreational parks can also be merged within one cluster due to very small differences in structure.

### Performance and comparison of study and public beds (within parks)

**Fig 7:**
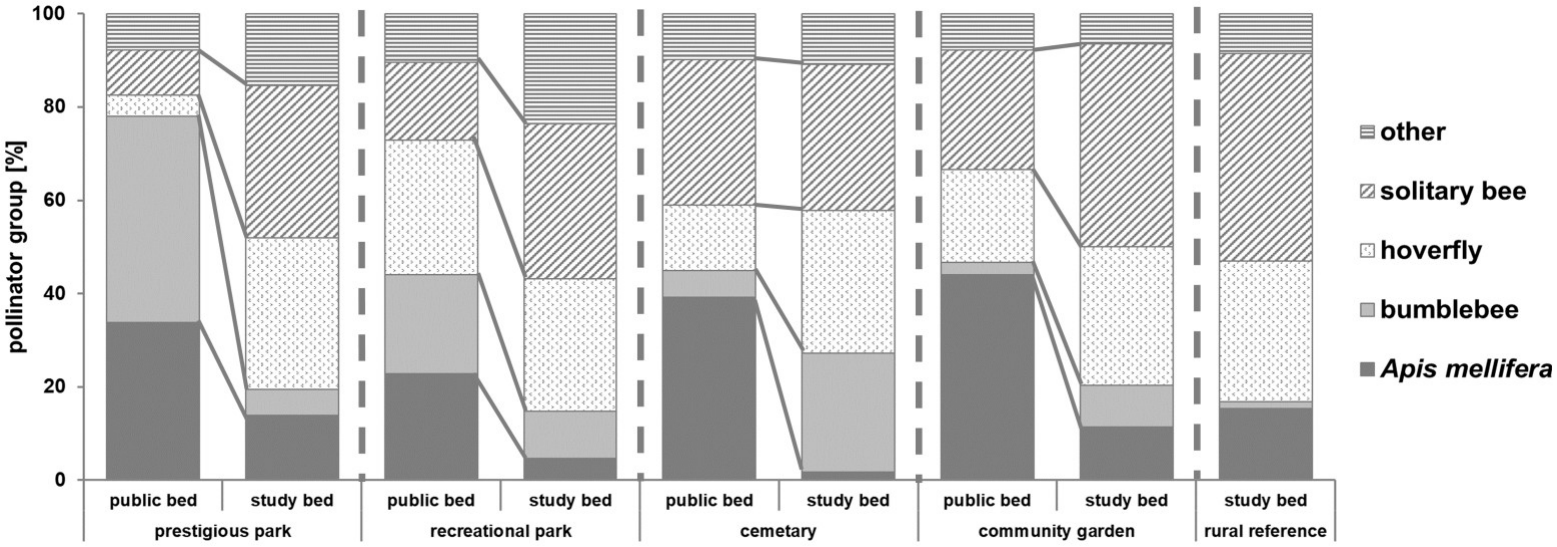
Percentage distribution of recorded individuals per pollinator groups in public beds and designed study beds for every type of green space. From black to grey bars: *Apis mellifera*, bumblebees, hoverflies, solitary bees and others.

A comparison of visiting pollinator groups between public beds and the designed study beds is given in Fig 7. The illustration of percentages was chosen to ensure direct comparability between study beds and public beds. The mean absolute abundances of the species groups measured on public beds (mean: 37.2) were significantly higher than on study beds (mean: 26.5).

Results of the percentage distribution show a general lower share of the eusocial colonizing insects bumblebees and honeybees in designed study beds compared to public beds. This result was evident for all types of parks. The percentage reduction of these two insect groups was between 18.0% (cemetery) and 58.7% (representative park). The amount of recorded *Apis mellifera* individuals was about 20% less in the representative park (33.8% to 13.9%), 18% in recreational parks (22.9% to 4.6%), 37.5% in the cemetery site (39.2% to 1.7%) and 32.6% in community gardens (44.0% to 11.4%). The percentage decrease of colonizing insects consequently led to higher proportional amounts of wild bees and syrphids.

### Plant-pollinator network analysis

**Fig 8:**
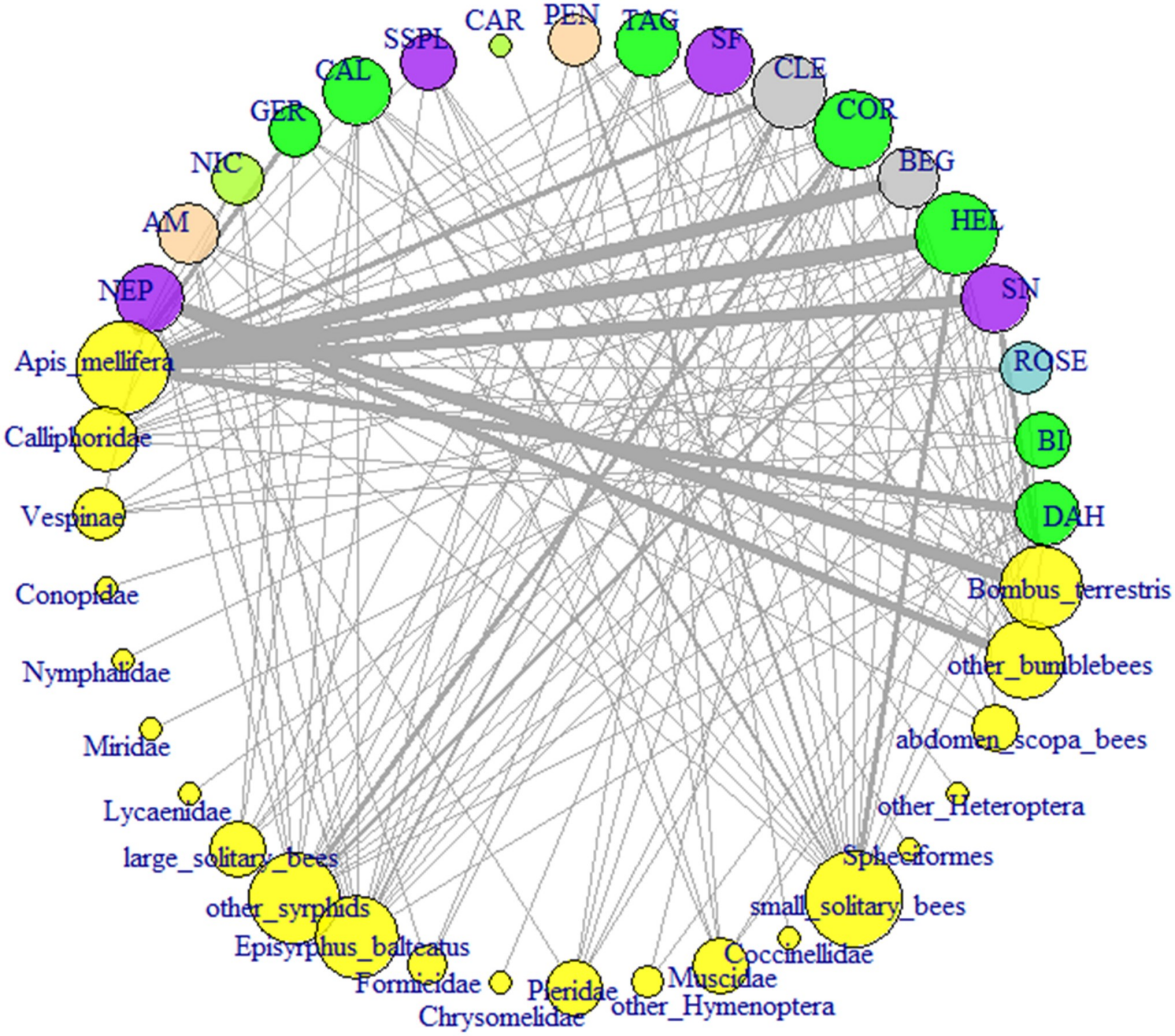
Plant-pollinator network of recorded plant visitations by pollinator groups. The color of plant vertices indicates the respective pollinating syndrome classification of the plants. Abbreviation of plants (pollen syndrome): COR: *Coreopsis lanceolata* (generalist); PEN: *Penstemon x gloxinioides* (bumblebee); AM: *Antirrhinum majus* (bumblebee); CAR: *Dianthus chinensis* (butterfly); CLE: *Tarenaya hassleriana* (n.d.); SF: *Salvia farinacea* (Hymenoptera); SSPL: *Salvia splendens* (Hymenoptera); DAH: *Dahlia hortensis* (generalist); CAL: *Calendula officinalis* (generalist); TAG: *Tagetes tenuifolia* (generalist); BI: *Bidens ferulifolia* (generalist); NEP: *Nepeta x faassenii* (Hymenoptera); ROSE: *Rosa x centifolia* (pollen); SN: *Salvia nemorosa* (Hymenoptera); GER: *Geranium x magnificum* (generalist); NIC: *Nicotiana x sanderae* (butterfly); HEL: *Heliotropium arborescens* (generalist); BEG: *Begonia semperflorens* (n.d.).

**Fig 9:**
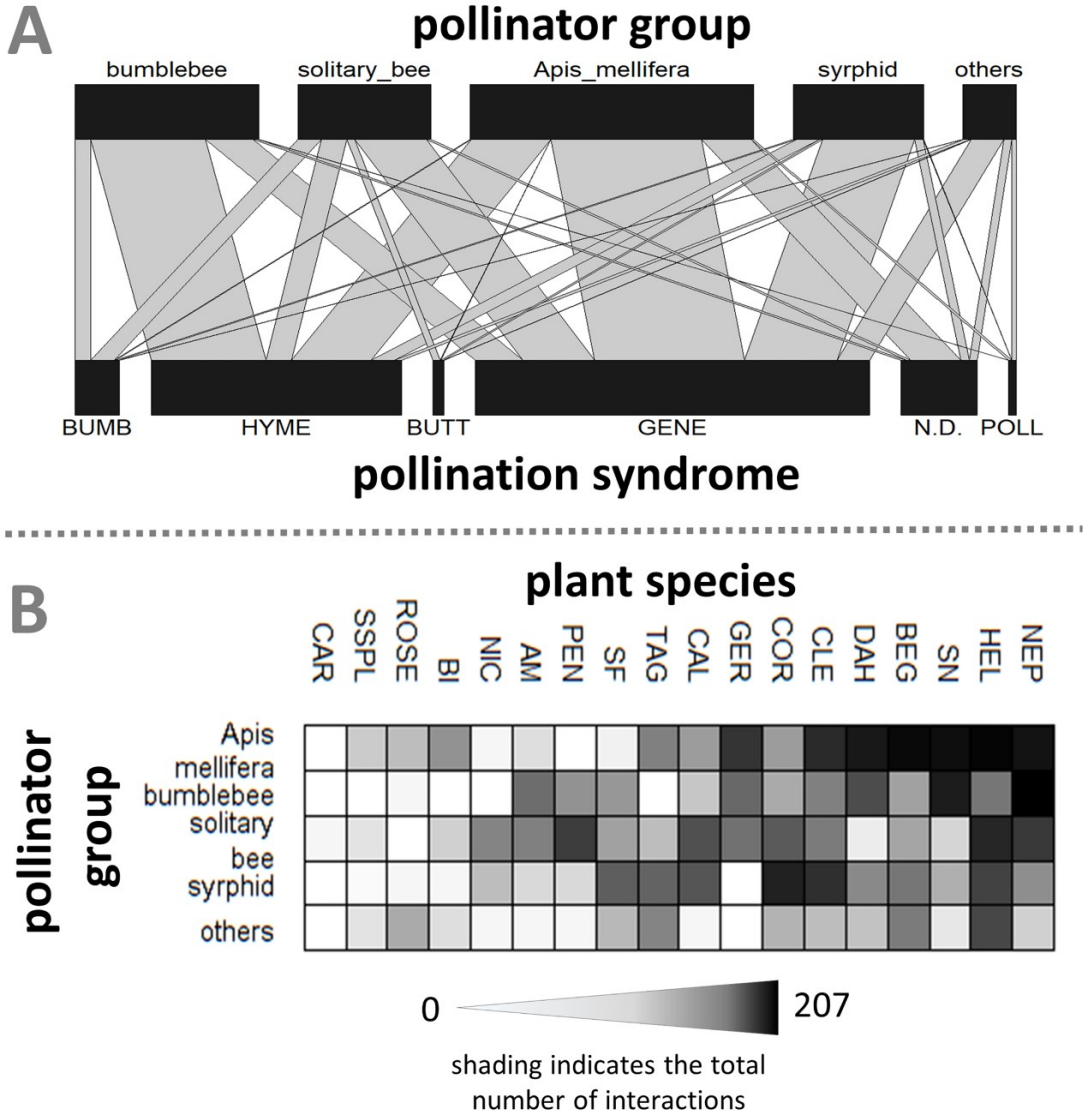
(A) Diagonal community interaction matrix (all plant species and grouped pollinators) and (B) the bipartite graph with aggregated pollinator groups and plants categorized according to their pollination syndrome. The shading of matrix entries indicates the number of observations. Abbreviation of plants (pollen syndrome): COR: Coreopsis lanceolata (generalist); PEN: Penstemon x gloxinioides (bumblebee); AM: Antirrhinum majus (bumblebee); CAR: Dianthus chinensis (butterfly); CLE: Tarenaya hassleriana (n.d.); SF: Salvia farinacea (hymenoptera); SSPL: Salvia splendens (hymenoptera); DAH: Dahlia hortensis (generalist); CAL: Calendula officinalis (generalist); TAG: Tagetes tenuifolia (generalist); BI: Bidens ferulifolia (generalist); NEP: Nepeta x faassenii (hymenoptera); ROSE: Rosa x centifolia (pollen); SN: Salvia nemorosa (hymenoptera); GER: Geranium x magnificum (generalist); NIC: Nicotiana x sanderae (butterfly); HEL: Heliotropium arborescens (generalist); BEG: Begonia semperflorens (n.d.); Abbreviation of plant pollinating syndrome: BUMB: bumblebee; HYME: hymenoptera; BUTT: butterfly; GENE: generalist; n.d.: not detectable; poll: pollen.

Results of the plant-pollinator network analysis for all pollinator groups and recorded flower species in public beds are shown in Fig 8. In Fig 9 the associated community interaction matrix and bipartite graph of the data set is presented. For this, single findings of pollinators were aggregated in one group. For the bipartite graph the plant species were classified into trait groups of different pollination syndromes. Calculated network metrics of all three levels of data aggregation are shown in Table 4.

**Table 4:** Network metrics of the calculated plant-pollinator networks for different level of data aggregation.

Network metrics	Network input data		
	All plant species All pollinators	All plant species Grouped pollinators	Group pollination syndrome Grouped pollinators
**level of data aggregation**	+	++	+++
**weighted nestedness**	0.576	0.040	-0.133
**connectance**	0.338	0.867	0.933
**interaction evenness**	0.676	0.828	0.750
**H**_**2**_**’ specialization**	0.233	0.201	0.153
**mean number of shared partners**:			
**pollinators**	2.2	13.9	5.2
**plants**	4.4	3.8	4.3
**Niche overlap**:			
**pollinators**	0.20	0.57	0.82
**plants**	0.51	0.56	0.49

The interaction pattern clearly illustrates the activity of a large number of different insect groups in urban green spaces, which act as pollinators and contribute to this ecosystem service. The total plant-pollinator network shows that a high diversity of interactions between pollinator groups and plants was recorded in the survey. However, it can also be noticed that not all pollinator groups visited the various plants, but that rather distinct plant pollinator groups were established. This is also shown in the community interaction matrix (Fig 9). The network illustrates a high degree of nestedness, which is primarily driven by the two colonizing species *Apis mellifera* and *Bombus terrestris*. Both species were particularly attracted by the flowering plants *Nepeta x faassenii* and *Salvia nemorosa* with a specific Hymenoptera pollination syndrome. Additional strong interactions were observed between *Apis mellifera* and plant species like *Begonia semperflorens*, *Heliotropium arborescens* and *Dahlia hortensis*, classified as generalist pollination syndrome.

The bipartite graph of the network (Fig 9) displays the distribution of insect visitations among different plant pollination syndromes. The vast majority of the visits of all insect groups took place on generalist pollination syndromes. Bees and bumblebees also showed a high level of interactions with plants of the Hymenoptera pollination syndrome. Visitations on flowers with a specialized pollination syndrome were observed comparatively rarely. However, it is apparent that visitations and pollination were mainly carried out by the pollinator groups that were expected on the plants due to the pollination syndrome. This is especially true for the bumblebee pollination syndrome.

Metrics of the plant-pollinator network support the visual impression of a nested assembly. The total network showed a relatively high weighted-interaction nestedness of 0.576 (1 = perfect nestedness) (Galeano et al., 2009) compared to the scores of the other calculated networks. In these aggregated network models, however, this pattern of nestedness is successively concealed by the accumulated data. The weighted nestedness was 0.04 for aggregated pollinator groups and -0.133 (= less nested than the corresponding random matrix) for plants additionally categorized into pollination syndromes. Same was true for the H_2_’ index, a measure of specialization that depends on interactions and marginal totals of species. High H_2_’ scores represent a high level of interaction selectiveness (compared to a null distribution of interactions) as a measure of network specialization [41]. The total network contained the highest degree of specialized interactions (0.233). This also led to a relatively low pollinator niche overlap (calculated as Horn’s index) of 0.20, compared to the pollinator niche overlaps of 0.57 and 0.82 for networks with aggregated data. The niche overlap for plants was relatively constant throughout the different network models (0.49–0.56 mean similarity).

The connectance estimator, which considers the realized proportion of possible links between plants and pollinators, showed as expected a contrary dynamic. In the most aggregated network almost all possible interactions between pollinator groups and classified plant species were realized (connectance index = 0.933). The total network only had about one third of the possible links between plant and pollinator groups.

### Visitations on Tilia trees

**Fig 10:**
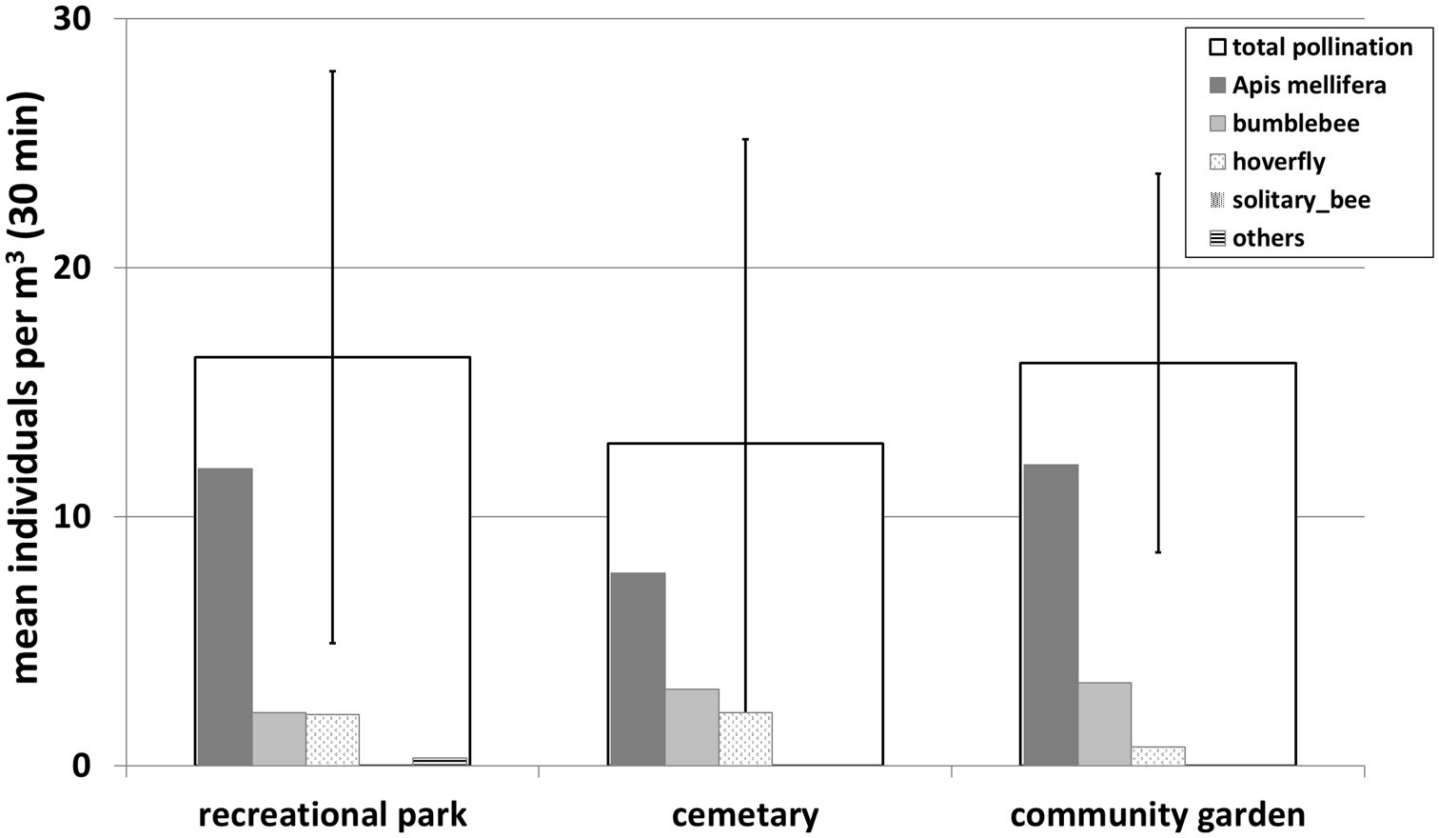
Mean number of recorded flower visitations in green volume of 1 m^3^ for Tilia trees (*Tilia* spp.). From white to black bars: Total visitations, *Apis mellifera*, bumblebees, hoverflies, solitary bees and others.

Recorded pollinator visitations in Tilia trees for different park types are shown in Fig 10.

The visitation rates of flowering Tilia trees showed similar frequencies for all tested park types. We found a mean number of 12.9 (cemetery) to 16.4 (recreational park) visits per 30 minutes and m^3^. In comparison to the visitation results in study beds (Fig 6) the compositions of insect species in Tilia trees had a different appearance. Visitors (and consequently the pollinators) of Tilia blossoms were dominated by *Apis mellifera* with 59.8% (cemetary) to 74.7% (community garden) of the recorded insects. Bumblebees (including *Bombus terrestris*) were also found regularly with 2–3 individuals per measurement. Syrphids were only recorded infrequently with a highest percentage of 16.5% in the cemetery. Other pollinator groups were only detected as single findings.

### Pollination performance within park types

**Fig 11:**
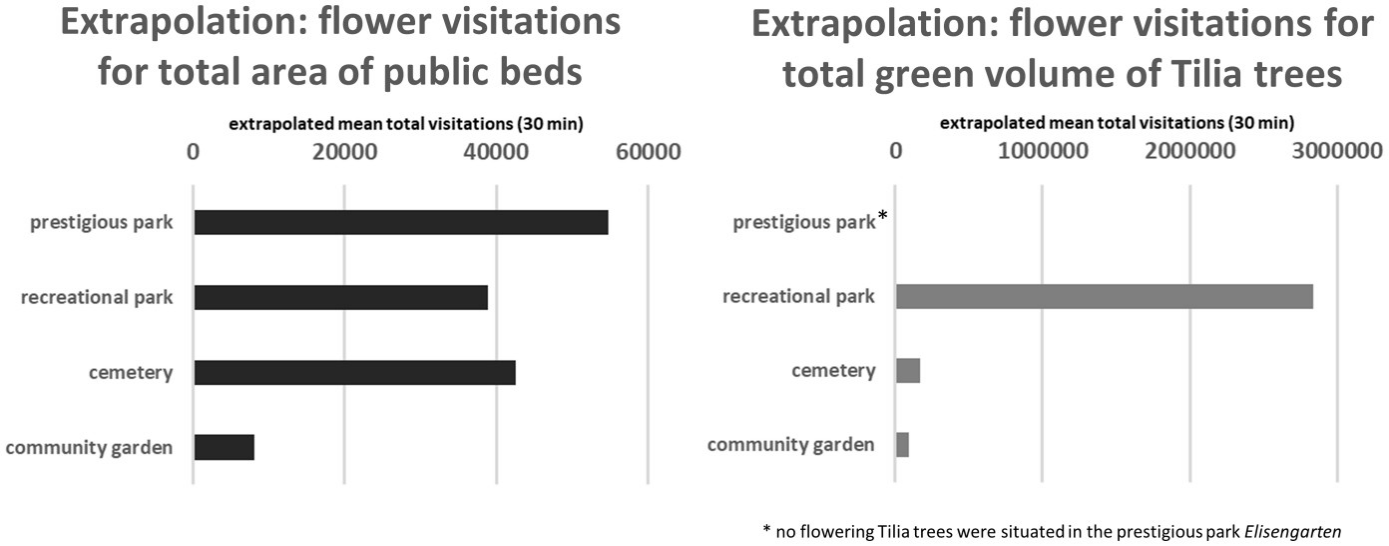
Extrapolation of mean total flower visitation rates for all public flower beds (left) and all insect-pollinated flowering Tilia trees (right) in the investigated park types of the city of Aachen, Germany.

The extrapolation of all flower bed and Tilia tree visitations by pollinating insects in the investigated green spaces in Aachen within 30 minutes are summarized in Fig 11.

For public beds, we calculated a total mean of about 144,000 insect visitations (*estimated value*) in 3504 m^2^ bed area within 30 minutes, summarized for the investigated green spaces in the city of Aachen under given environmental conditions (as described in section *sampling method*). Most of the bed visitations (about 55,000 *estimated*) were calculated for the prestigious park *Elisengarten* (1130 m^2^ flower bed area), followed by the cemetery *Ostfriedhof* (about 43,000 visits on 835 m^2^ bed area *estimated*) and the aggregated recreational parks (approximately 39,000 visits *estimated*, 1304 m^2^ flower bed area). The community gardens only showed a comparatively low mean number of total visitations in 30 minutes (8,000 visits *estimated*, 235 m^2^ flower bed area).

Tilia trees in the considered parks were visited in total approximately 3 million times within 30 minutes (*estimated value*). Due to a large occurrence of Tilia trees in the recreational parks, the visiting rate of the Tilia trees in this park type was enormously high (about 2.8 million visitations in 187,414 m^3^ green volume *estimated*). For the cemetery and the community gardens mean total visitations of about 165,000 (17,349 m^3^ green volume) and 93,000 (5,728 m^3^ green volume) were calculated (*estimated values*). In the prestigious park *Elisengarten* no insect-pollinating Tilia tree is located.

**Fig 12:**
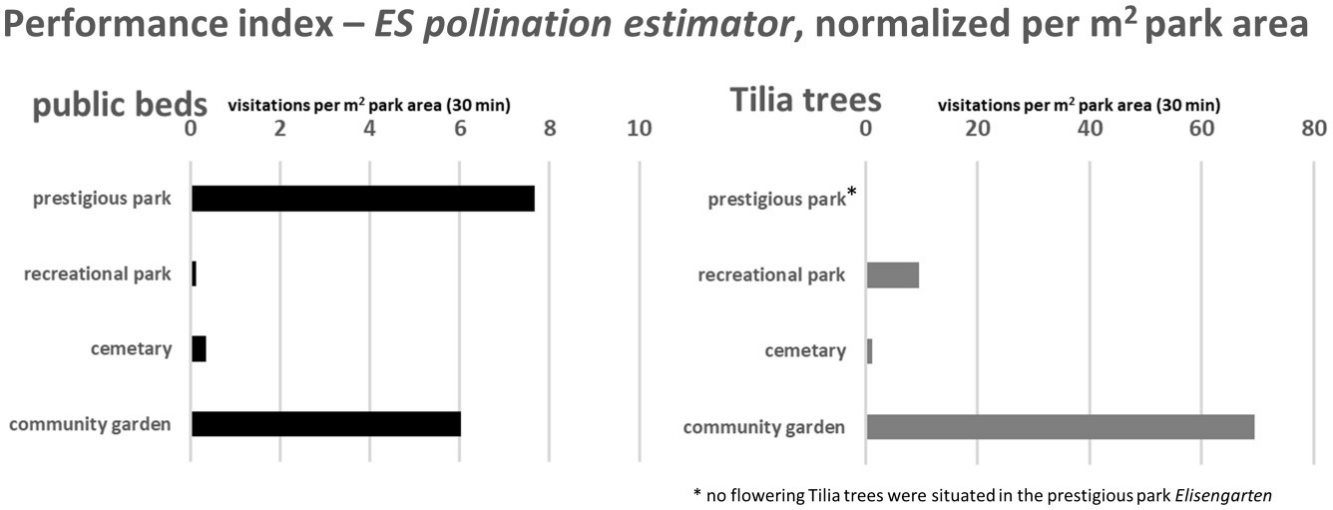
Calculation of the performance index (*ES pollination estimator*) for the supplied ecosystem service of pollination per m^2^ park area. The figures left (for public beds) and right (for Tilia trees) show an estimation of the park performance, expressed as mean total insect visitations per m^2^ park area.

Fig 12 illustrates an estimation to what extent the ecosystem service of pollination is supported by the structural composition of the park types. The total visitations were divided by the total surface area per park. The resulting *ES pollination estimator* was defined as a performance index for the ecosystem service of pollination in different types of parks.

With regard to a comparatively small total area of 1334 m^2^, the design of the community gardens with high percentages of insect-attracting flower beds led to a high-performing realization of the ecosystem service of pollination, as revealed by the *pollination estimator* of 6 visitations per m^2^ park area. This normalized index value was only exceeded by the prestigious park (7.7 visits/m^2^), where 15% of the total area is covered with flower beds (7,147 m^2^ park area). The percentages of flower beds in the recreational parks and the cemetery, on the other hand, are only 0.4% (298,648 m^2^ total recreational park type area) and 0.7% (125,000 m^2^ total cemetery area), which led to poor *pollination estimator* values of 0.1 and 0.3 visits per m^2^ park area. For Tilia trees, the highest visitation rates in relation to the total park area were calculated for community gardens (69.3 visitations/m^2^). Recreational parks had mean Tilia tree visitations of 9.5 visitations/m^2^, the *pollination estimator* of the cemetery was 1.3 visitations/m^2^.

## Discussion

In the following section, a comparative analysis of flower visitor communities between park types is presented, followed by the results of the pairwise comparison of the designed study beds and public park beds within each type of park. Furthermore, results of the plant-pollinator-network analysis and the pollination of Tilia trees are discussed. The extrapolations of total visitation rates as well as the realization of the ecosystem service provided by plant-pollinator interactions are critically reviewed, which ultimately leads to recommendations for the design of green infrastructure in cities.

### Comparison of flower visitor biocenosis between park types

The results of the study with designed beds showed that the design of the flower beds and the arrangement, management and setting of the surrounding green space can be decisive factors that influence the diversity and abundance of flower visitors.

We demonstrated that community gardens had different patterns and abundances of flower visitors than the other urban park types. They showed a very similar pollinator community structure as found in the rural reference (Fig 6). Numbers of recorded individuals per m^2^ study bed in community gardens and the rural reference were significantly higher than in other urban park types. Taking into account that the community gardens *Hirschgruen* and *Vielfeld* have only existed since a few years, the very dynamic potential of pollinator communities to colonize urban areas becomes apparent. The differences correlate with the alternative design and management concept of the community gardens. Compared to other urban green spaces, these green areas are characterized by a relatively high degree of near-natural, “pristine” plots and consist of small subareas that are mostly left to their own. In addition, small and diverse flower beds and meadows are designed.

The high flower density and diversity is a decisive factor, even more relevant for pollinator diversity than the habitat type [38]. As a result, particularly abundant and diverse communities with high numbers of solitary wild bees and hoverflies can develop and establish themselves. This provides a nature-based solution for pollination resources [42]. Accordingly, we recorded a similar pollinator community as observed in the rural reference equipped with extensively used meadows, which is the original habitat of many wild bees and syrphids in the cultural landscape. This illustrates the importance of urban community gardens for the pollinator community within the city of Aachen.

The result is in line with findings of previous studies, in which so-called substitution habitats in anthropogenic areas have already been noticed [51]. A suitable refuge was identified for species of solitary bees and their corresponding parasitic wild bee species, although some of them were considered to be extinct [52, 53]. Active protection management with little intervention on meadows in urban areas can also lead to colonization of endangered and specialized solitary bee species, as shown for the *Brander Wald Aachen* area [54].

The composition of the flower-visiting population in community gardens illustrates the high potential of cities to provide habitats for this crucial ecological group. We encourage to incorporate these insights to specify necessary *Leitbilder* on how urban green spaces could be designed to provide this ecosystem service accordingly (see section *Recommendations for policy action*).

The overall high ecological value with regard to the promotion of species richness was already shown in preliminary studies for structurally diverse and heterogeneous green areas [55]. Especially for urban community gardens Dennis and James [56] found synergistic effects between this ecological characteristic and social services (learning and well-being). Similar approaches to use urban green spaces like individually cultivated allotment garden plots have a comparable structural appearance. Thus, these gardens have a high potential to provide a range of various ecosystem services and functions in addition to the habitat potential for flower visitors and resulting ecosystem service of pollination [57].

### Comparison of designed study beds and public park beds within park types

Colonizing pollinator species with the ability to fly long distances, like *Apis mellifera* and *Bombus terrestris*, were recorded more often in public beds and less frequent in designed study beds. Public beds in urban green spaces are characterized by a significantly higher surface area than the small study beds of 1 m^2^. For this reason, a mass flowering resource is only established in public beds. This demonstrates that the large and monotonous pollination syndrome density is beneficial for pollinator with large foraging areas such as honeybees [58] and bumble bees [59], regardless of the respective green space concept and management of the park. Due to this foraging strategy these colonizing species are able to fulfil the comparably high nutrition level required for their hives.

On the small study beds, however, more solitary bees and hoverflies were found with a similar diverse range of pollination syndromes as on the public beds. The planting of small patches of green space can therefore serve as a food resource for various insect species and thus promote diversity at one location. Small areas can be used to create a flowering supply that is attractive for other pollinators than the large public beds. Both the design of the parks and the sociality of the pollinator groups considered have an impact on the occurrence and distribution of species.

The pairwise comparison of designed study beds with the respective public beds illustrates that the designed beds do not attract the same patterns and abundances of flower visitor communities. The pollination of flower-constant, colonizing insects is reduced in study beds, despite the design of various flowering syndromes. Therefore, these beds are supposed to be more able than monotonous public beds to indicate the diversity of communities in the immediate surroundings as a habitat potential of these green spaces. This consequence of flower constancy in combination with behavioural flexibility of competing pollinator species has already been discussed in detail in previous studies [60]. It is therefore highly informative to carry out investigations of all pollination syndromes occurring in parks to analyse the diversity of the pollinators' communities. Comparative studies of only designed beds cannot be used to compare actual pollination performance in the parks, since conclusions on the total diversity of species and abundance of individuals cannot be drawn. To assess the state of plant-pollinator-interactions within a city, it would also be highly informative to compare it with more pristine sites like meadows. These should be located in the same natural region and can thus serve as development objectives.

### Plant-pollinator network analyses

The calculated plant-pollinator network of study data emphasizes the differences in behaviour between the observed colonizing pollinator groups and the solitary bees and hoverflies. While social insects showed rather a high flower constancy, i.e. visited the same plant species at high frequencies (Fig 8), many other plant species were visited by a multitude of different solitary pollinators. Thus, a mutual interrelation of certain pollinator groups only with specific flower types can be observed. Insect flower constancy is described for a wide range of pollinators [61–64] and was already observed for honeybee workers in the year 340 BC by Aristotle [65].

This mechanism is also depicted in the calculated network metrics of the plant-pollinator interactions. In our statistical analysis it can be recognized by the weighted nestedness estimator that considers interaction frequencies and which was proposed by Galeano, Pastor [66]. The relatively high weighted-interaction nestedness (0.576) for this urban network was also observed for plant pollinator interaction analyses in other landscapes and settings [67]. The nested character of plant-pollinator interactions was demonstrated to increase the structural stability of pollinator communities, perturbation of plant or pollinator community results less often in extinction of species population [68]. This indicates a stable composition of the pollinator community, a presumably good connectivity to non-urban surroundings and consequently a stable re-colonization potential of new green spaces in the city. In our study this was found for the relatively new designed community gardens.

On species level we found a high level of weighted nestedness in conjunction with a low connectance index, which illustrates the possible links between plants and pollinators within the network. For urban plant-pollinator communities this results indicates, that the visitation and pollination behaviour of pollinators has a non-random character. Thus, the design of beds and green spaces has a major impact on insect population within cities. According to the mutualistic relationship, planting of flowering plants with high diversity of species and pollination traits will increase functional diversity and structural stability of pollinator communities. As a feedback effect, this will lead to a more diverse urban plant community [42] assuming that spontaneous vegetation within the urban area is granted. Cities have the potential to serve as a reservoir of functional pollinator diversity within increasingly degraded cultural landscapes by providing habitats for stable pollinator communities. This may be crucial for a sustainable provision of the ecosystem service of pollination.

Our comparison of the plant-pollinator network with different level of data aggregation for plants and pollinators clearly showed that the nestedness of the network increased with network complexity. This complexity is given by an increased number of flower groups, pollinator groups and their interactions. This characteristic of plant-pollinator networks could already be determined by Bascompte, Jordano [67]. It reveals that a comparison of the network metrics across different plant-pollinator networks requires the same level of aggregation, generally at species level.

Nevertheless, the network analysis based on plant pollination syndromes, i.e. taking the pollination trait of plant species into account, demonstrates that this distinct plant classification does not always withstand empirical data: plants with bumble bee syndrome (*Penstemon x gloxinoides* and *Antirrhinum majus*) are often visited by solitary bees. Both plants have large perianths and corolla tubes, which is ideal for foraging bumble bees but does not exclude pollination by solitary bees.

For pollinators, results are more evident at plant trait level. Especially the Hymenoptera and generalist syndrome showed the expected large variety of different pollinating insect groups. For the recorded plant-pollinator network in the city of Aachen, however, this clustered approach does not result in a sufficiently resolved representation of plant pollinator interactions that would allow further conclusions to be drawn about interaction dynamics. The plant-pollinator network shows a much stronger significance at species level.

### Tilia pollination on investigation sites

Results of blossom visitations show that flowering Tilia can play a key role as food sources of colonizing insects like *Apis mellifera* and *Bombus terrestris* in cities. Even if the mean total visitation rates of all pollinator groups in one m^3^ of Tilia treetop are slightly lower than in a m^2^ flower bed, the overall visitations of Tilia trees in investigated parks are more than 20 times higher during vegetation period than for flower beds. High number of flower visitations in Tilia trees by foraging bees were already recorded in previous investigations [69] which underlines the importance of insect-pollinating trees in cities [70].

Insect-pollinating trees are especially relevant for eusocial flower visitors, since the trees are highly dominant as a food source in urban green spaces and have a high nectar content, i. e. social flower visitors can store more nectar during the flowering period. For solitary bees, small and constant flowering is especially important because they do not store so much food.

Up to now there is no scientific evidence for the longstanding assumption that nectar of Tilia trees, especially of *Tilia tomentosa*, has toxic effects on bumble bees and honeybees [71]. The observed frequent mass mortality close to Tilia, especially of bumble bees, results from starvation due to insufficient food resources after the late flowering of Tilia trees within the vegetation period [71]. Silver linden have the latest flowering period of planted Tilia species in the city. After that period, foraging insects only find few food sources, mainly in flower beds. Honeybee colonies are rather able to compensate this gap due to stored nectar, which is one of the reasons why individuals of *Bombus terrestris* are the most frequent observed insect species under Tilia trees.

For this purpose, the colonizing insects need a continuous supply of insect-pollinating flowers in the remaining flowering period in order to overcome the gap in nectar flow of trees [7, 71]. This again demonstrates the high relevance of the city beds in time periods with insufficient nectar and pollen supply by trees.

### Total pollination service in urban parks

The prediction of total visitation rates and pollination performances in parks, based on insect records and surface areas of park elements, leads to valuable insights regarding the relevance of structural elements or park types for the ecosystem service of pollination. In our study, the recreational parks in Aachen show comparably less visitation rates and a low pollination service performance. These park types have a particularly high potential to improve conditions for pollinators with an adjustment of the supply of flowering beds and insect-pollinating trees.

The low percentages of flower beds in recreational park and, consequently, very low *pollination estimator* values for this park types clearly showed, that this type of green spaces have a huge potential to increase food supply and habitat conditions for the pollinator community in the urban environment (see section *Recommendations for policy action*). The *pollination estimator* as an indicator value for the provision of park structures to support the ecosystem service of pollination clearly illustrates, that the community gardens in Aachen are managed in a way that improves habitat quality for pollinating insects in the city. Results of total visitation rates also confirm that Tilia trees have a key role as food sources in cities during the vegetation period, compared to total visitation rates of flower beds.

Nevertheless, the calculation of visitation rates and park performances regarding the total pollination services in the city of Aachen should only be interpreted as rough estimations, due to the use of mean abundance values for calculating flower visitations and the *pollination estimator*. For this reason, only rounded extrapolation values were presented in the result section. The high variability in visitation rates throughout the observations was not considered in this calculation (Fig 6). In addition, we extrapolated pollination performances from one investigated square meter in each observation. Even if we made a lot of effort to select a representative type of public bed for the respective park type, the number of blossoms and blossom visitations might be varying for other beds within the park types. Therefore, it is important to stress that the estimated values of this extrapolation step do not provide precise information on actual, total flower visitations. It should be used as a coarse approximation of the order of magnitude of pollination and as a comparison of these scales between park types. Moreover, we can only interpret the *pollination estimator* as an indicator regarding total visitation rates of pollinators, a conclusion regarding the diversity of pollinators at a site is not included and has to be analyzed separately.

The calculation of overall pollination can also serve as a rough estimation for the total pollination performance of the city of Aachen, because we investigated all major green spaces in the very city center. Nevertheless, we assume that private gardens, roadside greenery and vegetation close to railway tracks, streams and rivers have a major impact on pollination in cities. To assess this, a comprehensive survey of relevant vegetation elements in cities (beds, hedges, insect-pollinating trees) is mandatory. An automatic categorization of park structures by using remote-sensing techniques and data could help to close this gap.

### Discussion of methodology

In the study the pollinator communities were recorded during the peak season from May to August. However, since various insect groups are foraging and pollinating at different time periods of the year, the study should be continued to fully record the pollinator community during the entire vegetation period.

Regarding the plant-pollinator interaction analysis, our comparison of network metrics between different level of data aggregation emphasizes the importance of high quality ecological data when assessing the ecological properties of urban green spaces:

Highly aggregated data, which may have been recorded inaccurately or with poorly resolved taxonomic determination level may disguise or distort patterns in community structure. A meaningful derivation of underlying processes is no longer possible. In the calculated network interaction analysis, the connectance estimator for highly aggregated data illustrates an almost complete realization of the possible interactions of plants and pollinators. Compared to this, the total network on species level reveals that pollinators actually specialize. Not all pollinator species visit and pollinate all different plant species. In consideration of these findings and with the endeavour to work as non-intrusively as possible, it is crucial to identify and record flower visitor species (or morphological groups) as accurately as possible at the investigation site. This requires a high degree of experience and expert knowledge in the identification of insect species in the field. Even then, a complete determination at species level is often not feasible [72]. By taking this into account the selected recording method, which is similar to methods carried out in previous studies [25, 37], leads to meaningful results.

For the extrapolation of pollination services by calculating the *pollination estimator*, we used the data on visitation rates to assess the quantity of pollination. Nevertheless, we cannot make any statements regarding the quality of the actual process of pollination, as these can considerably differ among insect species [73]. For a more accurate assessment of pollination, this aspect requires empirical investigations and an integration into the *pollination estimator* as a weighting parameter.

## Conclusions and recommendations for policy action

Our results indicate the strong potential of cities to provide a diverse habitat for different groups of pollinators. It is necessary to rely on near-natural concepts in design and maintenance and to even use the smallest green patches to improve the availability of food sources and nesting habitats for flower visitors as well as the pollinator diversity itself. Most recommendations can be put into practice on different levels of organisation: politically on regional assembly and local council level, practically by management practices of park departments in city municipalities, and by communities and societies like allotment associations and private citizens.

In order to promote habitat quality and pollination increased planting of native, insect-pollinated flowers and trees in public parks of types is recommended [43, 74]. Especially recreational parks have a high amount of unused potential in terms of food sources. In artificial beds, it is recommended to plant regional seed mixtures and plant compositions [23, 47]. Even small micro-habitats with a diverse planting can be used as pollinator food supply as they are quickly inhabited by different flower pollinators [31], especially wild bees and hoverflies. By supplying food sources for a diverse pollinator community plant-pollinator interactions are enhanced and the loss of ecosystem functions reduced [75, 76].

In the context of urban apiculturing activities [77] increased planting of insect-pollinated trees is recommended. An insect-friendly composition may include time-delayed flowering Tilia species (*T*. *platyphyllos*, *T*. *cordata*, *T*. *tomentosa*, especially as a food source for *Apis mellifera*) but also *Acer pseudoplatanus*, *Aesculus carnea*, *Aesculus hippocastanum* and *Robinia pseudoacacia* [70]. This could reduce the risk of too large temporal gaps of food sources within a vegetation period and the incipient mass death of eusocial species like *Bombus terrestris* [71].

We recommend to generate more structurally diverse green spaces by reducing the level of management interventions, as spontaneous vegetation and more natural areas provide opportunities for various insect habitats. Self-organization of ecological processes and consequently resilience of the ecosystem and its services (like pollination) will be supported by the development of self-regulated structures [78–80] like nesting facilities. Preservation of even small-scale unplanned areas should be integrated into planning guidance to promote local biodiversity, especially for solitary wild bees and syrphids. On a city scale level, we also recommend to reinforce the realization of regulation services by promoting functional and structural connectivity between green infrastructure [81], facilitating the movement of insects.

Since these ecosystem service-focused recommendations cannot be considered separately in multifunctional cities, they have to be combined with other functions and services that are expected and desired by urban green spaces for citizens [82]. We thus advocate an integrated management of urban free spaces, considering parks as key habitats for pollinators in anthropogenic dominated environments and finding so-called *Leitbilder* for urban green spaces in sustainable cities [83]. As already stated by Baldock, Goddard [31], the improvement of the value of growing urban landscapes for pollinators should be a key component of national strategies for the conservation and restoration of pollinator communities.

## Supporting information

S1 Table Visual examples of the differentiated park types in the research study. (PDF)

S2 Table Classification of pollinator genus and species into flower visitor categories of the research study. (PDF)

S3 Table Plant species and respective pollination syndrome of public flower beds in green spaces in the city of Aachen, Germany, during summer 2016. (PDF)

S4 Table Pre-studies for testing and adjustment of the chosen study design. (PDF)

S5 Table Raw data of conducted field study. (CSV)
