# Supplementary material for: A “plan bee” for cities: Pollinator diversity and plant-pollinator interactions in urban green spaces
Source: PLoS One. 2020 Jul 15;15(7):e0235492. doi: 10.1371/journal.pone.0235492 (PMC7363068; doi:10.1371/journal.pone.0235492)
Supplement: S1 Table — (PDF) [file pone.0235492.s001.pdf]

S1 Table: Visual examples of the differentiated park types in the research study.

Prestigious park	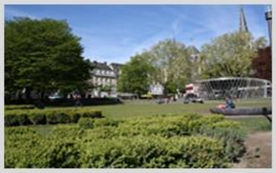
Recreational park	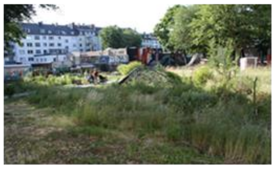
Cemetery	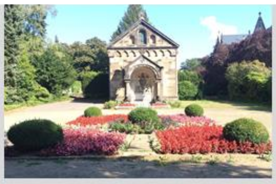
Community garden	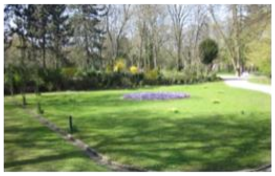
Rural reference	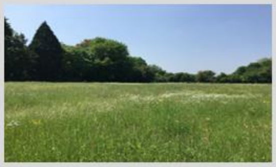
