# Supplementary material for: A “plan bee” for cities: Pollinator diversity and plant-pollinator interactions in urban green spaces
Source: PLoS One. 2020 Jul 15;15(7):e0235492. doi: 10.1371/journal.pone.0235492 (PMC7363068; doi:10.1371/journal.pone.0235492)
Supplement: S2 Table — (PDF) [file pone.0235492.s002.pdf]

S2 Table: Classification of pollinator genus and species into flower visitor categories of the research study.

Morphological groups in general survey (designed flower beds, trees)	classification for plant-pollinator interactions (public beds)	associated pollinator genus and species
**Apis mellifera**	*Apis mellifera*	western honey bee *Apis mellifera*
**bumblebee**	*Bombus terrestris*	large earth bumblebee *Bombus terrestris*
	other bombus species (incl. Psithyrus)	e.g. *Bombus hortorum, Bombus lapidarius, Bombus pratorum, Bombus hypnorum, Bombus pascuorum*
**syrphids / hoverfly**	*Episyrphus balteatus*	marmalade hoverfly *Episyrphus balteatus*
	all other Syrphidae	e.g. *Eristalis, Dasisyrphus, Helophilus, Platicheirus, Volucella, Xylota, Syrphus*
**solitary bees**	small solitary bees	mainly small species of *Halictus, Lasioglossum &* *Hylaeus*, but also small Megachilidae like *Chelostoma, Osmia & Heriades*, 6-10 mm length
	large Megachilidae (sternit scopa bees)	large members of the family *Megachilidae* e.g. *Megachile* (leafcutter bees), *Osmia* (mason bees), *Anthidium* (carder bees)
	large solitary bees	other wild bees > 10 mm, e.g. *Andrena*, *Colletes*, large *Halictus & Lasioglossum*
**others**	Calliphoridae	-
	Formicidae	-
	Chrysomelidae	-
	Pieridae	-
	Vespinae	-
	other_Hymenoptera	
	Muscidae	-
	Pentatomidae	-
	Lyniphiidae	-
	Conopidae	-
	Nymphalidae	-

	Spheciformes	-
	Tipulidae	-
	Polistinae	-
	Xiphydriidae	-
	Chrysididae	-
	Coccinellidae	-
	Berytidae	-
	Agelenidae	-
	Miridae	-
	Noctuidae	-
	Lycaenidae	-
	Pyrrhocoridae	-
	other_Heteroptera	
