# Supplementary material for: A “plan bee” for cities: Pollinator diversity and plant-pollinator interactions in urban green spaces
Source: PLoS One. 2020 Jul 15;15(7):e0235492. doi: 10.1371/journal.pone.0235492 (PMC7363068; doi:10.1371/journal.pone.0235492)
Supplement: S3 Table — (PDF) [file pone.0235492.s003.pdf]

S3 Table: Plant species and respective flower syndrome of public flower beds in green spaces in the city of Aachen, Germany, during summer 2016. If no information about flower syndrome of a concrete species was available, the information was taken from a related sister species (http://floraweb.de/ , www.biolflor.de). A representative of the morphogroup is shown and marked in bold.

flower syndrome	Example of flower syndrome	Species (correction factor for plant pollinator network analyses)	site
**Hymenoptera syndrome**	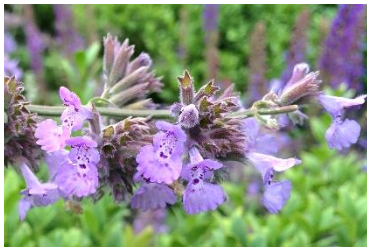	***Nepeta x faassenii* (1.08)**	Elisengarten
		Salvia nemorosa (0.67)	Elisengarten, Frankenbergpark
		Salvia farinacea (1.08)	Stadtgarten
		Salvia splendens (1.08)	Stadtgarten
**pollen syndrome**	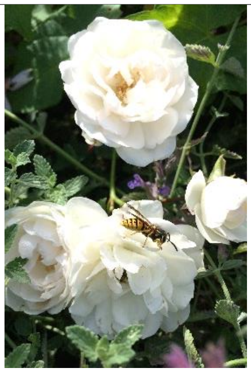	*Rosa x centifolia* (1.08)	Elisengarten
**generalist syndrome**	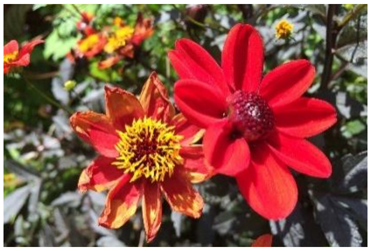	*Geranium x magnificum* (1.08)	Frankenbergpark
		Bidens ferulifolia (1.08)	Community garden Hirschgrün
		Calendula officinalis (1.08)	Community garden Hirschgrün
		Dahlia hortensis (0.52)	Community garden Hirschgrün, Vielfeld
		Tagetes tenuifolia (1.08)	Community garden Hirschgrün
		Heliotropium arborescens (1.08)	Ostfriedhof
		Coreopsis lanceolate (1.08)	Westpark
**butterfly syndrome**	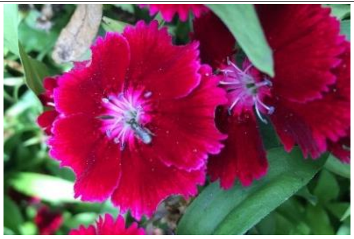	*Nicotiana x sanderae* (1.08)	Ostfriedhof
		Dianthus chinensis (1.08)	Westpark

**bumblebee syndrome**	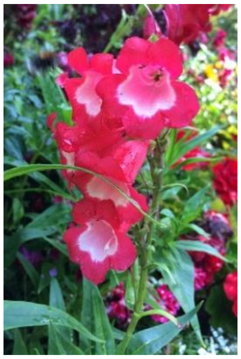	*Antirrhinum majus* (1.08)	Westpark
		Penstemon x gloxinioides (1.08)	Westpark
**n.d.**	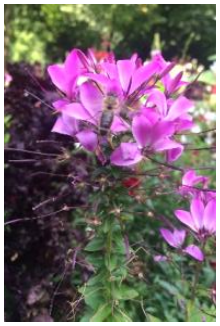	*Begonia semperflorens* (1.08)	Ostfriedhof
		Tarenaya hassleriana (1.08)	Westpark
