# Supplementary material for: A “plan bee” for cities: Pollinator diversity and plant-pollinator interactions in urban green spaces
Source: PLoS One. 2020 Jul 15;15(7):e0235492. doi: 10.1371/journal.pone.0235492 (PMC7363068; doi:10.1371/journal.pone.0235492)
Supplement: S4 Table — (PDF) [file pone.0235492.s004.pdf]

S4 Table: Pre-studies for testing and adjustment of the chosen study design; s=number of different investigated structural units (=number of different beds/trees).

year	Park type	Structure type	Number of samplings	N total
**2015**	Representative park	Public bed (s=1)	21	120
	Recreational park	Public bed (s=5)	69	
	Cemetery	Public bed (s=1)	30	
**2014**	Representative park	Public bed (s=1)	30	102
	Recreational park	Public bed (s=5)	18	
	Community garden	Public bed (s=1)	27	
	Rural reference	Public bed (s=3)	27	
			**$N_{pre\text{-}studies}$=**	**222**
